# Negotiating the boundary between medicine and consumer culture: Online marketing of nutrigenetic tests^☆^

**DOI:** 10.1016/j.socscimed.2009.10.066

**Published:** 2010-03

**Authors:** Paula M. Saukko, Matthew Reed, Nicky Britten, Stuart Hogarth

**Affiliations:** a Department of Social Sciences, Loughborough University, Brockington Building, LE11 3TU Loughborough, Leicestershire, UK; b University of Gloucestershire, Cheltenham, UK; c Peninsula Medical School, Universities of Exeter and Plymouth, UK; d King's College, London, UK

**Keywords:** Genetics, Science and technology studies, Boundary work, UK, USA, Nutrigenetics, Direct-to-consumer genetic testing

## Abstract

Genomics researchers and policy makers have accused nutrigenetic testing companies—which provide DNA-based nutritional advice online—of misleading the public. The UK and USA regulation of the tests has hinged on whether they are classed as “medical” devices, and alternative regulatory categories for “lifestyle” and less-serious genetic tests have been proposed. This article presents the findings of a qualitative thematic analysis of the webpages of nine nutrigenetic testing companies. We argue that the companies, mirroring and negotiating the regulatory debates, were creating a new social space for products between medicine and consumer culture. This space was articulated through three themes: (i) how “genes” and tests were framed, (ii) how the individual was imagined vis a vis health information, and (iii) the advice and treatments offered. The themes mapped onto four frames or models for genetic testing: (i) clinical genetics, (ii) medicine, (iii) intermediate, and (iv) lifestyle. We suggest that the genomics researchers and policy makers appeared to perform what Gieryn (Gieryn, T.F. (1983). Boundary-work and the demarcation of science from non-science: strains and interests in professional ideologies of scientists. *American Sociological Review, 48*, 781–795.) has termed “boundary work”, i.e., to delegitimize the tests as outside proper medicine and science. Yet, they legitimated them, though in a different way, by defining them as lifestyle, and we contend that the transformation of the boundaries of science into a creation of such hybrid or compromise categories is symptomatic of current historical times. Social scientists studying medicine have referred to the emergence of “lifestyle” products. This article contributes to this literature by examining the historical, regulatory and marketing processes through which certain goods and services become defined this way.

## Introduction

Genetic testing has traditionally been managed by clinical geneticists concerned with rare and often incurable disorders, such as Huntington's disease, caused by a single gene. The test results have informed sensitive reproductive decisions, which might be facilitated by specialist counselling.

The recent major public, private and charitable investment in genomic research—including vast projects, such as the Human Genome Project and the UK Biobank—has been justified by the promise that genetic tests will benefit the population at large. Both UK (Department of Health, 2003) and USA (Khoury, 2003) government agencies have envisioned that soon primary care clinicians will offer most people genetic tests for susceptibilities for common diseases together with preventive advice and treatments.

A number of small biotech companies have gone further and sold genetic tests – such as paternity and ancestry tests, tests for susceptibilities for illness or addiction or whole genome scans – online. In this article, we focus on a subset of these companies, which offer nutrigenetic tests, i.e., a service whereby customers send DNA samples (mouth swab) and receive, often via mail, advice on diets and supplements based on testing of a few gene variants. Following the sociology of technology literature (Pinch & Bijker, 1989), we examine nutrigenetic tests as a technological artifact in the making or before it becomes taken-for-granted or fails. Examining such emergent technologies highlights how they are shaped by diverse social actors. We are especially interested in how nutrigenetic testing companies shape their products on their online marketing and sales portals. Yet, the online marketing only makes sense against the background of the wider network of actors interested in the technology.

There were only a handful of nutrigenetic testing companies in 2008, but they had attracted considerable public attention. The media had often more or less advertised the companies, an example being the 2007 ITV (a UK independent television channel) programme *The Killer in Me*, which featured four celebrities taking a test offered by Genetic Health and claimed to “open a window not just on their lives but potentially their deaths” (The Killer in Me, 2007; on the industry bias in media coverage of nutrigenetics see Caulfield, Shelley, Bubela, & Minaker, 2009).

But nutrigenetic testing companies have also attracted negative attention. The critics of the companies have included non-governmental organisations, such as the UK GeneWatch, but most of the disapproval has come from individuals and institutions involved in genomics research and policy. Much of the contention has focused on the scientific validity of the tests, with critics accusing the companies in popular, scholarly and regulatory forums of selling “snake oils” (Pollack, 2006). GeneWatch questioned the principle of providing dietary advice based on genetic tests, stating in its parliamentary briefing soon after Sciona started marketing its nutrigenetic test in the UK that “genes are poor predictors of complex diseases” (GeneWatch, 2002). Most critics were more circumspect. Dr. Muin Khoury and colleagues from the U.S. Office for Genomics and Disease Prevention, Centers for Disease Control, which was in charge of developing ways to use genomic research to prevent disease, stated in the prestigious journal *Nature Genetics* that the science of using genetics to enhance healthy lifestyle “holds great promise” but was “not ready for prime time” (Haga, Khoury, & Burke, 2003: 350). Similarly, in 2006 investigators from the U.S. Government Accountability Office (GAO) bought a series of nutrigenetic tests and accused them of making “medically unproven” and “meaningless” predictions, whilst hedging their critiques with statements on how “genetic testing is becoming an integral part of healthcare with great potential for future test development and use” (GAO, 2006).

Overall, the debate on nutrigenetic testing resembled what Gieryn has termed “boundary work”, i.e., “ideological efforts by scientists to distinguish their work from non-scientific intellectual activities” (Gieryn, 1983: 782). In the case of nutrigenetic testing scientists and functionaries, frequently associated with genomics research and/or policy, sought to distinguish responsible and promising future genomic science and medicine from irresponsible nutrigenetic companies, who had launched the technology prematurely. However, due to many critics' vested interest in genomic research and policy they contradictorily buttressed the promise of the technology whilst deriding nutrigenetic tests. Only GeneWatch questioned the principle of using genetic tests as a basis for preventing common diseases.

The question of what should be done about nutrigenetic tests also became muddled in the regulatory realm. Nutrigenetic testing companies had been able to sell their products due to a loophole in regulation, which did not require “home brew” laboratory tests to undergo review by Food and Drug Administration (FDA). In 2006 in hearings in front of Senate's Special Committee on Aging, following the damning GAO report, the issue became whether nutrigenetic tests were medical devices, and if so, which risk classification they should belong to. As stated by Dr. Steve Gutman from FDA during the hearings:

A product is a medical device if it is intended for diagnosis of disease or other conditions, or for use in the cure, mitigation, treatment, or prevention of disease. To the extent the tests GAO investigated make such claims; they are devices subject to FDA jurisdiction (Gutman, 2006: 2).

In response Rosalynn Gill-Garrison, Sciona's Chief Technology Office testified in the same hearings that the company's services were not, indeed, medical but offered:

… genetic information to consumers concerning their nutritional wellbeing, contributing to their health and wellness. Sciona is not involved in diagnostic or disease-related services or information (Gill-Garrison, 2006).

In the UK, the regulation of genetic tests was governed by the European Union's In Vitro Diagnostic Devices (IVDD) Directive, which was implemented nationally by the Medicines and Healthcare Products Regulatory Agency (MHRA). Similar to the USA situation, the regulation hinged on the question of whether nutrigenetic tests would fall under the ambit of the IVDD Directive as “devices used for medical purposes,” and if so how their risk would be classified. The Human Genetics Commission (HGC) – an advisory body consisting of individuals with a genetics interest, such as clinical geneticists, genetic counselors, scientists, patient and industry representatives and members of public, such as science journalists – had been pushing for the regulation of direct-to-consumer (DTC) genetic testing since 2003 (HGC, 2003). In its 2007 report on DTC-testing, HGC noted that MHRA had notified them that “so called ‘lifestyle’ tests” do not fall under their remit (HGC, 2007: 16). The commission reported that it had considered recommending that all genetic tests should be regulated in the same manner but had concluded it would be “a mistake.” Rather, it ended up recommending that …

… an alternative regulatory mechanism be created to tests, such as ‘lifestyle’ tests, which are currently not regulated as they fall outside the scope of the IVDD directive (HGC, 2007: 16).

Due to legal technicalities and the prevailing view among the genomics community that not all genetic tests were alike (i.e. “new” genetic susceptibility tests were qualitatively different from the old single gene clinical genetic tests, e.g., Khoury, 2003), the regulatory debates were creating a new regulatory and marketing category for non-medical or “lifestyle” genetic tests. Further, even if the tests would be classified as medical, in the EU genetic tests would be considered low risk, and if this was to be the case in the USA they would not require FDA premarket review. Within this emergent framework, nutrigenetic tests were falling into possibly several categories between medical and consumer products, such as lifestyle or low-risk medicine, and be expected to fulfill some, yet to be specified, criteria.

As stated by an industry representative during pilot fieldwork in 2006, in this situation the companies needed to choose a strategy between developing products more akin to “diet drinks and skin creams,” which customers do not necessarily expect to work, or more serious “cardiovascular products,” which would need to be backed up by more extensive research. Different companies were choosing slightly different strategies. For example, an industry representative stated that nutrigenetic tests “should” be classed as medical devices and regulated in a similar manner to disease risk and pharmacogenetic tests. However, the representative acknowledged that …

… there would be industry pressure to separate [nutrigenetics], to allow companies that are not going to specialise in the area we're doing to be a bit freer with their you know, eat lots of broccoli and take some of our own homemade pills type thing (Interview with an anonymous industry representative (P26) in June 2008).

In this statement, the industry representative was also doing boundary work (Gieryn, 1983) in an attempt to distinguish his company, willing to comply with more stringent regulation for medical devices, and companies selling lifestyle genetic tests and “homemade pills.” The statement also illustrates how different companies were pitching their products to slightly different marketing and regulatory niches between lifestyle and light-medicine.

In this article, we will analyse the webpages of nutrigenetic testing companies as a case study of the production of a new marketing and regulatory category between medicine and consumer culture. We will also pay attention to how different companies shape the category differently.

### Biomedicalisation and consumerization

The undertakings of the nutrigenetic testing companies may be fascinating per se, but we were interested in them because they were symptomatic of broader transformation in medicine. They also highlighted a few rarely discussed or conceptualised features in this transformation.

As already discussed, the efforts of representatives of public policy and genomic research to construct a boundary (Gieryn, 1983) between proper science and medicine and nutrigenetic testing in the popular, scholarly and regulatory realms were not entirely successful. Gieryn's work has frequently been used by social scientists to make sense of the strategic actions of scientists to exclude others in, for example, stem cell research (Wainwright, Williams, Michael, Farsides, & Cribb, 2006). There is a paucity of studies on cases where boundary work has faltered. Further, many of Gieryn's (1999) case studies focus on the 19th century when science and medicine were delineating their autonomous professional spheres of expertise against competing authorities, such as organized religion and industrialists. It has been argued that the boundaries between science/medicine and varied industry, government and lay interests have recently blurred (e.g. Nowotny, Scott, & Gibbons, 2001). This development is likely to render boundary work increasingly tenuous. We contend that the transformation of defending boundaries of science into a creation of hybrid or compromise categories, such as lifestyle genetic testing, is symptomatic of current historical times. It also remains a poorly researched area.

Medical sociologists have made sense of current, historical transformations in medicine with terms such as biomedicalisation (Clarke, Shim, Mamo, Fosket, & Fishman, 2003) and information-based medicine (Nettleton, 2004). Clarke et al. (2003) argue that biomedicalisation is characterised by increasing privatisation and commodification of medical research and services, which expands biomedical jurisdiction into ever new areas of life. Studies on processes of (bio)medicalisation have focused, for example, on how phenomena such as shyness, sadness (Conrad & Leiter, 2004) or impotence (Fishman, 2004) are attributed a biological/molecular cause (also Rose, 2007), assigned a diagnostic label (social anxiety disorder, depression, sexual dysfunction) and a concomitant pharmaceutical product (Paxil, Prozac, Viagra).

Nutrigenetic testing is similar to these cases in that it biomedicalises an everyday phenomenon – eating – by associating it with a molecular underpinning and specific diets or dietary products. The contribution of our nutrigenetics case study to this literature is that it highlights how biomedicalisation and attendant privatisation and commodification happened through the creation of a new marketing and regulatory space for “lifestyle” products between medicine and consumer culture. Gieryn's (1983, 1999) notion of boundary work helps to make sense of the historical specificity of this development. Even if the media and regulatory debates appeared as boundary work, they did not delegitimize nutrigenetic tests as outside science/medicine, as happened to, for example, phrenology or composting in Gieryn's (1999) historical cases. Rather, the debates created new rules that legitimated the tests on slightly different grounds.

The casual use of the term “lifestyle” or “enhancement” in relation to nutrigenetic tests is common not only in regulatory texts but also in social science research literature (e.g. Geransar & Einsiedel, 2008; Martin & Frost, 2003) and is also used, for example, to describe Viagra (Fishman, 2004; Fox, Ward, & ÓRourke, 2005) and over-the-counter cholesterol lowering statins in the UK (Edgley, 2007). However, these works use the label in a descriptive manner; they do not examine how its recurrence across scholarly, regulatory, media and everyday realms creates a new social space for such products. This is what we will investigate.

## Methods

To explore how nutrigenetic testing companies were shaping the technology amidst regulatory and public debates, we analysed their webpages, on which they marketed and often directly sold the tests. We analysed the webpages of all companies marketing nutrigenetic tests on the Internet in 2007 and 2008. The sample is complete to the best of our knowledge, given the difficulty of tracking down frequently changing websites. The companies analysed were: Genelex (USA), Genovations/Genova Diagnostics (USA), Genetic Health (UK), Interleukin Genetics/Quixtar (USA), Market America (USA), Nutrigen (USA), Salugen (USA), Sciona (USA, formerly UK), and Suracell (USA).

The forces behind nutrigenetic testing companies included scientists; for example, one of the managers of Genetic Health, Dr. Paul Jenkins was a Reader in endocrinological oncology at the University of London. Many of the companies had complex connections with the consumer industry. The largest investor in Sciona was DSM, specialising in nutrition, pharmaceuticals, plastics and chemical products (DSM, 2006). Nutrigenetic testing companies also sold consumer products, which accounted for the main source of revenue for Interleukin Genetics (Interleukin Genetics, 2008), which had a commercial partnership with the consumer company Alticor/Amway, proprietor of brands, such as Nutrilite™. Many companies did not sell their own tests. Genelex and Market America distributed Sciona's test under a different name. Genelex marketed a number of other DNA tests, Market America sold a range of consumer products from supplements to garden furniture; between 2006 and 2008 Sciona also sold its tests through varied and changing intermediaries, such as grocerystores and holistic pharmacies. The UK Genetic Health sold the test by the Austrian company Genosense. Overall, this complex alliance of science and consumer, pharmaceutical, retail and wellness/alternative medicine industry was well poised for navigating the regulatory and marketing landscape for lifestyle products.

To provide an overview of the products sold by the nutrigenetic testing companies, we have described in Table 1 what tests and associated products they sold and how they purported to deliver them. In analyzing the webpages, we only focused on marketing material for nutrigenetic tests and associated products (supplements) and tests (cardiovascular tests), not unrelated tests (paternity testing) or consumer goods.

The webpages of the companies were multimodal (Lemke, 2002) and hypertextual or intertextual. This means that they combined textual material with visuals (images, layout etc.) and provided their audiences also with links, which directed visitors to press coverage of the company, to scientific articles as well as company responses to public criticism of their services. The webpages, thus, were targeted not only at customers but also at investors, distributors, scientists, regulators and public media.

We first printed a selection of the companies' webpages in 2007. Three authors (PS, MR, NB) read the material and made note of frequent themes and images. We followed the principle of constant comparative method (Glaser, 1965), which seeks to inductively identify recurrent themes in the material. After developing a preliminary coding scheme all the texts on the current webpages were downloaded into NVivo 7.0 qualitative software and coded by MR and PS in 2007 with further revisions to the pages added and coded by SH in spring 2008. We also took notes of visuals in conjunction with the texts on the webpages and analysed them informed by constant comparison (see Clarke, 2005).

We then undertook further analysis of how different companies gave the themes slightly different spins, clustering into a few “frames,” following Goffman's (1974/1986) classic work and its application to media analysis (Gamson & Modigliani, 1989). Frames structure experience or suggest “what is at issue” (Gamson & Modigliani, 1989: 3); they are produced by strategic actions of individuals and groups, but in order to be successful they have to resonate with contemporary social sensibilities, and they are often produced through subtle clues that suggest the meaning of the issue, for example, whether it is serious or humorous, i.e., a fight or a play-fight (Goffman, 1974/1986: 41–44). In media, texts frames are produced through depictions, catchphrases or catchwords and visual images (Gamson & Modigliani, 1989: 3), and we analysed how the frames in our material operated through these devices.

## Results

### Main themes

Three prominent themes emerged from the analysis of the companies' websites: (i) the nature of genes and the tests, (ii) the role of the individual vis a vis genetic information, and (iii) the advice and treatments offered. The companies articulated the three themes slightly differently, framing their products as “lifestyle,” “medicine,” or in-between the two (“intermediate”). Based on this observation, and our knowledge of the controversies around the tests, we constructed a stratified classification scheme for genetic tests—ranging from clinical genetics through to lifestyle genetics—which was taking shape on the webpages and in the debates. We located the companies' services and promotional strategies within this scheme (see Table 2). In what follows, we will analyse how these frames were articulated on the webpages.

### Genes, illness and wellness

On their webpages, many nutrigenetic testing companies explicitly distanced their services from clinical genetic testing:

Can you tell me if I carry the genes for a serious illness?

No. The Gene SNP DNA Screening Analysis is not a test for inherited disorders or inherited predisposition to disease. We do not screen for disorders caused by a defect in a single gene, such as Huntington's disease, cystic fibrosis or sickle cell anemia. (Market America, 2008, FAQs).

Through such statements, the nutrigenetic testing companies distinguished their services from clinical genetic tests for disorders “caused” by a “single gene” and perceived to be riddled with ethical conundrums. In so doing, they mobilized the distinction frequently underlined in genomics research (Khoury, 2003) and regulation (HGC, 2002: 52–54) – paving the way for introducing genetic testing into everyday healthcare – between “serious” single gene tests and susceptibility tests with “intermediate” or “low” “impact” (HGC, 2002). The nutrigenetic tests were thus framed as “less serious” than clinical genetic ones, suggesting they were to be consumed and regulated more lightheartedly or liberally.

When it came to defining the exact nature of the nutrigenetic tests, the companies had carefully crafted slightly different descriptions. Some companies (especially Sciona and its distributors Genelex and Market America) went to great lengths to avoid associations between their services and diagnosis or prediction of disease:

Optimize the health of your skin and bones; heart and mind by optimizing your personal diet and supplement intake. Genetic testing combined with a lifestyle assessment, provide you with a scientifically based, personal blueprint for optimizing health (Genelex, 2008, nutrigenetic test home).

The choice of the catchphrase, “optimize health,” reflected the regulatory debates, discussed previously, seeking to frame the tests as not “medical devices” but lifestyle. Further, the references to “health of your skin and bones,” did not bring into mind avoiding illness but enhancing beauty and fitness or strength. The combination of words “heart and mind” in relation to health does not immediately evoke the physical organ heart but ambiguous mental and physical wellbeing. One only needs to imagine how different the statements would read if words, such as “osteoporosis” or “cardiovascular disease,” were used. This illustrates how the “lifestyle” frame was created not just by avoiding disease-related claims with legal consequences but through consistent clues that suggested the tests were not serious medical devices but ambivalent wellness products (see Goffman, 1974/1986: 41–44).

Some companies, such as Salugen, combined catchwords associated with medicine and those associated with lifestyle or consumer culture, which is why we have termed their framing “intermediate.” Often times the catchphrases producing the intermediate frame were contradictory or even odd. An example is Salugen's description of the “timeline” of genetics innovations from Gregory Mendel and Watson and Crick until the initiation of the Human Genome Project in 1986 and…

2005—Salugen pioneers *nutritional gene therapy* by analyzing a person's DNA to customize their nutritional supplement pill ingredients (Salugen, innovations timeline, 2008, emphasis in the original).

The italicized catchword here is inherently contradictory. The word “nutrional” referred to nutrients and eating, i.e., lifestyle or consumer culture. “Gene therapy” generally refers to a distinctly serious and experimental technology of modifying “defective genes” into healthy ones and has been, largely unsuccessfully, used to treat life-threatening conditions, such as forms of human immunodeficiency. Combining these words created a confusion as well as suggested that nutritional therapies may modify genes, i.e., that genes are not fixed. The convoluted term “nutritional supplement pill ingredients” is similarly contradictory, “nutritional” and “ingredients” often associated with food, i.e., consumer culture, “supplements” with lifestyle or wellness products and “pills” with medicine, but in a colloquial sense. This statement illustrates how the intermediate frame frequently mixed lifestyle and medical frames, producing intentionally and sometimes clumsily confusing definitions and statements.

Some nutrigenetic testing companies (particularly Genetic Health, Genovations and Interleukin Genetics) framed their services as akin to medicine, also spelling out the catchwords disease and prediction.

Our predictive genomic profiles assess genetic variations in each person that, when combined with modifiable factors in the environment, may increase disease risk. This empowers physicians and patients to realize:

• Earlier, more effective preventive interventions-years before disease develops;
• Precise, customized therapies that truly address each individual's needs;
• Improved clinical insight into patients with treatment-resistant “chronic” conditions (Genovations, 2008, introductory page).

In this paragraph, Genovations indicated it assessed “disease risk,” which could signal to regulators that it was selling medical devices. It also used clinical words, such as physicians, patients, interventions, treatments and chronic conditions, throughout the text. Even if the statement did not say much concrete about the company's tests or treatments, the ubiquitous clinical vocabulary framed the services as serious or more like “medicine” rather than consumer culture.

The three frames (lifestyle, intermediate and medicine) were also created through the use of visuals and colours. In 2007, Sciona's frontpage featured three images on white background: one of a youngish smiling couple, against a green, leafy background, one of gleaming sweet peppers and one of a scientist in white suit and goggles peering into a microscope. These stock photographs sought to associate Sciona with lifestyle (the couple, the peppers) and science. Salugen's frontpage in 2008 featured a large photograph of an extended family, sitting on a shore in informal summer-clothes. The image showed their bodies from the neck down (as if to protect anonymity) and next to each person a caption stated, for example, “predisposed susceptibility to diabetes.” Whilst the clothing of the people and the setting communicated informality, the captions created an ominous sense of illnesses passed on in the family. The frontpage of the UK company Genetic Health had a white background with light green accents and featured a small image of a stethoscope on the side and a gleaming lime in the company banner. Whilst the stethoscope and the airy and rather formal and professional layout framed the company as “medical,” the image of the lime and light green softened this frame, communicating lifestyle or wellness.

Overall, the nutrigenetic testing companies dissociated themselves from clinical genetic tests for genes that “cause” disease. They were creating new frames or models for genetic tests as either lifestyle or less-serious medicine or somewhere between the two. The companies created these categories not only through explicit statements about not being medicine but consistent framing of their tests as more informal than medical devices through rhetoric, images and colour-schemes on their webpages.

### Between self-health and patients

Another prominent feature of the webpages was the way in which they addressed their customers as an individual, who should take personal charge of her/his wellbeing:

Whether you choose to “see” your genes or not, they are always there … By choosing to look at them, you are giving yourself the opportunity to do something about them. In this way, you can more actively—and more accurately—promote your health (Genovations, 2008, consumer page, patient's guide to genomics).

This statement depicted the customers of the tests as individuals who should acquire personalised, genetic information to improve their health or prevent disease. This depiction reiterated and was supported by the ubiquitous, contemporary individualist or neo-liberal preventive public health agenda—of which preventive genomics forms a part—according to which individuals should improve their personal behaviour to prevent common diseases (e.g. Department of Health, 2001; Harvey, 2009; Rose, 2007).

All companies depicted their customers as active health-seekers, but they differed in terms of how they configured the users of their services and how they delivered those services (see Woolgar, 1991 on configuring users of technology). Companies framing their tests as lifestyle (Sciona and its distributors Genelex and Market America) all sold their tests online, although Sciona also offered them through varied intermediaries. Sciona configured its customers as making sense of their health using a graph, combining results from the genetic test panel and lifestyle questionnaire:

For each of the health categories, your current position on the MyCellf Position Map-identified by the icon “You Are Here”-is plotted by the combination of your Gene Assessment (which cannot change) and your current Diet and Lifestyle Assessment (which can change by following the recommendations suggested in your personalized report). Your optimal health goal is identified by the “Your Goal” icon (Sciona, 2007, action plan samples).

Sciona's description of its map and the exemplary, colourful map displayed on the webpage constructed health/illness not as “lesions in the body” but as informational simulation (see Nettleton, 2004). The user of the Sciona's services was, thus, configured as acquiring personalized, virtual information about his/her health online and modifying his/her behaviour according to the simulation on their own. This stands in stark contrast with the traditional doctor-patient relationship, based on the former's expert look and touch in “a clinic.”

The webpages, dotted with sections such as details on products and payment methods, also configured the purchaser of the genetic test as any online shopper. At its barest, this normalcy was evoked by Market America's catalogue, which displayed a simple cut-out image of the nutrigenetic test kit package together with the price and “add to cart” icon, indicating it was like any consumer product sold on the portal.

The companies framing their products as in-between medicine and consumer culture (“intermediate”), such as Suracell, Salugen and Nutrigen, framed their customers vis a vis their services slightly differently. They emphasized that they offered the tests in quasi-medical settings, such as spas and wellness centers, aided by professionals of more or less ambiguous qualifications, such as “registered nutritionists” (Nutrigen) or trained lifestyle or aging “coaches” (Suracell):

Why is the Suracell Personal Genetic Health Program offered through Medispas, Physician's Offices, and Wellness Centers? These types of facilities are best able to provide a favorable atmosphere for genetic health through recommending stress reduction, proper diet and exercise. Personnel at these facilities often have strong backgrounds in age management, and Suracell provides them with training in the science of Personal Genetic Health (Suracell, 2008, frequently asked questions).

The references to spas, wellness, stress reduction and favorable atmosphere configured the customer as being served in a pleasurable setting or being “pampered.” The attention provided for the customer was depicted as professional but not medical with references to expertise, training and background in stress reduction and age management. Yet, Suracell, Nutrigen and Salugen all at some point between 2006 and 2007 sold their tests direct online. So, the references to relaxation and anti-aging also configured the customer as purchasing online products associated with beauty and health rather than medicine. This notion was reinforced by images, such as Salugen's large depiction of a young, nude woman immersed in water (on its SpaGen page), symbolizing rejuvenation and sensuality and frequently employed in advertising for personal hygiene and cosmetic products.

Companies framing their tests as predicting disease risk (Genetic Health, Genovations) indicated on their webpages that their tests were available through a physician. For example:

Preventive genetic diagnostics helps to explain why individuals are affected differently by the same environmental factors. Most importantly, it enables the physician to select suitable measures for his patient tailored to his individual genetic needs (Genetic Health, 2008, doctors' area).

In this description, users were configured as “patients” in a traditional doctor-patient relationship, where the expert makes the decisions in a paternalistic fashion, such as “selects suitable measures.” The users were also addressed indirectly, as the pages were formally addressed to physicians (“doctors area”) communicating that the company was selling the tests to healthcare professionals. The expectation of a clinical relationship was supported by images of stethoscopes (Genetic Health) or of a middle-aged, male doctor reading notes (Nutrigen). Regardless of these depictions, Genetic Health offered its nutrigenetic tests direct online. On its webpage, the company noted it can “email [the test-]report directly to you” but “recommended” that “one of our doctors go through it with you,” “on the telephone” or in “our clinic” (Genetic Health, 2007, DNA testing services). Genetic Health, thus, did not necessarily configure its customer as a patient in a clinic but as someone purchasing a medical-like service online but backed up with clinical support, if required, via phone or face-to-face.

The online sale of nutrigenetic tests was often raised in regulatory discussions. Nevertheless, even if proclamations were made about the need for professional guidance, the recommendations frequently veered towards regulation of information provided to the consumers (e.g. Gutman, 2006), suggesting a consumer regulation rather than a medical one. The UK Human Genetics Commission had made a rather woolly recommendation that “specific tests” should be offered through “specific outlets” (HGC, 2007: 3), indicating that the delivery of genetic tests should be arranged, seemingly on a sliding scale, based on the nature of the tests. In this situation, the companies were tallying the way they framed their tests with how they delivered them. So, companies that framed their services as lifestyle, delivered their tests direct online and companies that had framed their tests as medicine indicated on their webpages that their customers would receive, in some form, advice from a physician.

Similar industry self-regulation has been observed by Fox et al. (2005), who discovered that drugs, such as Viagra and Orlistat (for weight loss), which were considered “lifestyle” were sold in UK online pharmacies. Fox et al. (2005) did not explore how certain drugs became defined as “lifestyle,” as we have done. Yet, their research suggests that pharmaceutical and consumer industry—responding to regulatory pressures and seeking to create a market—were strategically creating a new category of “less serious” or lifestyle products between medicine and consumer culture, which could be sold in more liberal ways. The sale of nutrigenetic tests in conjunction with computer-generated, “personalised” graphs, in spas, and with phone consultation with a physician illustrated what the liberal ways of delivery might mean in the future, not only in relation to genetic tests but “lifestyle” services more broadly.

### Food, formulas and drugs

An important component of the nutrigenetic testing companies' marketing were descriptions of the advice or treatment offered based on the genetic test. The companies' profiles also differed in this respect.

Sciona, in particular, focused on offering advice on diet and foods:

First, make your choices from a variety of whole foods each day, preferably organic whole foods. Click here to read What about whole foods? Whole foods retain all their original nutrients, in the correct proportions intended by Mother Nature (Sciona, 2008, Healthy Living pages).

In this statement, Sciona emphasized the “naturalness” (with references to organic foods and Mother Nature) of the treatments it offered. This description accomplished two things. First, it mobilized the ubiquitous marketing discourse for things “natural” that frequently evoke notions of wholesome past and non-Western cultures to sell varied products from perfume to fair-trade coffee beans (on the colonialist underpinnings of such marketing see Stacey, 2000). In relation to health, the naturalness of foods also gains its meaning from its opposition to “artificial” drugs, and people have been found to prefer natural remedies, such as plant-based supplements, to pharmaceuticals, which are considered potentially dangerous, habit-forming and “unnatural” (e.g. Nichter & Thompson, 2006). Second, by offering healthy eating advice on the back of the nutrigenetic tests Sciona circumvented accusations of giving medical advice and appeared offering advice that was harmless. Indeed, in its report on DTC genetic testing Human Genetics Commission noted that the nutrigenetic tests and advice were “fairly innocuous” (HGC, 2007: 3).

However, the innocuousness of the advice also became a target of criticism. In the GAO report, nutrigenetic testing companies were accused of offering “common sense” advice, such as recommending eating vegetables, as allegedly genotype-specific (GAO, 2006). This critique accused nutrigenetic testing companies of giving advice that was too general, failing to meet the traditional expectations of medicine to identify a single cause and fetch a “silver bullet” treatment to target it, as in bacteria/antibiotics. Many nutrigenetic companies, particularly those framing their products as between medicine and lifestyle or “intermediate” (Nutrigen, Suracell, Salugen), but also others (Genelex, Market America, Interleukin Genetics) had solved the problem of providing treatments that were too general or too medical by offering various supplements or “formulas,” purportedly befitting a specific genotype. Interleukin Genetics's description of its patented “CardioEA^®^” formula illustrated the typical contents of such formulas:

The doctor recommended 81 mg dose of Aspirin with the combination of Vitamins B6, B12, Folic Acid, L-Arginine and Aged Garlic Extract^®^” for “heart health” (Interleukin Genetics, 2008, Innovations).

These formulas constituted quasi-medicines – often containing vitamins, plant extracts and compounds possibly having health benefits – between food items and drugs. The way in which the formulas were described and concocted also strategically associated them with “natural” remedies, such as garlic and other plant compounds, and medicine, such as Aspirin.

Still, a few nutrigenetic testing companies indicated on their webpages that they would provide advice about “medical” treatments:

The knowledge of the personal genomic constellation of a patient helps the physician to select suitable primary preventive therapeutic measures as well as to plan individual medication in order to avoid or reduce specific risks of developing CVD [cardiovascular disease] (Genetic Health, 2007, doctors' area).

Here Genetic Health, referring to medications, indicated that its physicians would provide advice about pharmaceuticals in association with the genetic test. In so doing, the company represented itself as providing “proper” or serious and specific interventions, rather than general dietary advice or ambiguous quasi-medicines. At the same time, it also made the company liable to accusations of “unnecessarily” medicalising people in light of the questionable validity of the tests, which has been raised by genomic researchers (Melzer & Zimmern, 2002) and regulators (HGC, 2003). The choice of “treatments” foregrounded the general dilemma facing the companies of whether to offer potent but potentially serious/harmful products or ambiguous but harmless ones.

The regulatory initiatives focused on the nutrigenetic testing technology and treatments and advice were only referred to in terms of the potentially harmful “effects” of the tests (HGC, 2007: 2). There already existed special regulation for products offered by the nutrigenetic testing companies, such as supplements and nutraceuticals. The Dietary Supplement and Health Education Act of 1994 had forbidden manufacturers to make claims about “health and disease” but it did not require them to strictly “prove” their claims (Nichter & Thompson, 2006). The case of functional foods, such as cholesterol lowering spreads, had been slightly different in that FDA had required the manufacturers to submit evidence for their claims but had allowed them to claim a health or disease benefit (Lehenkari, 2003). This regulation where vague claims provoke less stringent regulation and vice versa parallels the way in which nutrigenetic testing companies were pitching their products and services slightly differently. As nutrigenetic companies had close connections with the nutrition and consumer industries, it is conceivable that they had used the marketing and regulation of nutritional products as a model in designing a strategy for the genetic tests. The novelty of nutrigenetics was that it encompassed an entire family of liminal products from diagnostic tests to matching treatments, perhaps giving a preview of how the commercial health risk identification and prevention market will develop in the future.

## Conclusion

The little research published on nutrigenetic testing so far has focused on the poor quality of information on the companies' webpages (e.g. Geransar & Einsiedel, 2008; Stirling, 2008), although it has also been suggested that the field resonates with the contemporary, individualist preventive health agenda (Harvey, 2009). Others have looked for ways to regulate nutrigenetic testing, suggesting differential categories for “consumer and lifestyle” and “predispositional” testing (Martin & Frost, 2003) or arguing against the notion of lifestyle genetics (Melzer et al., 2008).

We have not evaluated the webpages against a standard or reflected on how the information and products offered by nutrigenetic testing companies should be regulated. Rather, we have examined how these scholarly, regulatory and popular discussions were creating a new social space for products between medicine and consumer culture, which was unraveling on the companies' websites.

The existence of markets for consumer goods with healing qualities is, obviously, not new (see Tomes, 2001). What we claim is new, or at least this development has become much more intense, is that medical/research, policy and industry communities are creating a new regulated space for such liminal products. The historical specificity of the development is highlighted by Gieryn's (1983, 1999) research on boundary work. The genomics researchers and policy makers in public, scholarly and regulatory forums appeared to be performing boundary work (Gieryn, 1983, 1999), i.e., to delegitimize the tests as outside of proper medicine and science. Yet, distinctly different from Gieryn's historical cases, the commentaries ended up creating a new marketing and regulatory space for the tests between medicine and consumer culture, which legitimated them, even if in different terms.

Sociologists have argued that contemporary medicine and science are losing their 20th century autonomy and become infused with commercial, government and lay interests. This has been described by terms such as biomedicalisation (Clarke et al., 2003) or mode 2 science (Nowotny et al., 2001). We contend that the emergence of categories of products between medicine and consumer culture is one of the mechanisms through which the boundaries of medicine/science blur.

Social scientists have noticed the emergence of “lifestyle” products, such as commercial genetic tests and drugs (e.g. Edgley, 2007; Fishman, 2004; Fox et al., 2005; Geransar & Einsiedel, 2008). Yet, they have not studied how these products became defined this way. There is also scattered research that discusses the creation of new self-(regulatory) categories for online pharmacies (Fox et al., 2005), dietary supplements (Nichter & Thompson, 2006), functional foods (Lehenkari, 2003) and over-the-counter cholesterol lowering statins (Edgley, 2007). This research suggests a surge of products with an ambiguous status vis a vis medical and consumer goods. Our article is taking this emergent research area forward by analyzing the regulatory and marketing processes that seek to create an identity and open up a social space for these products.

We have argued that the regulation of nutrigenetic tests was ambiguous part because the novel tests fell between the cracks of existing regulation. But the contradictory comments and recommendations made by genomics researchers and policy makers about the tests was also influenced by the fact that they shared the companies' interest in promoting genomic applications and the contemporary individualist self-health agenda. This illustrates how the new regulation was facilitated by the specific interests of genomics research and policy community and the broader individualist or neo-liberal zeitgeist, which forged the commercial, government and scientific agendas behind the new category.

We have pinpointed how the companies mirrored and negotiated the regulatory positions by associating their services with health, medicine and science but framing them (Goffman, 1974/1986) as more “informal” than medical products. Such framing was consistently applied through rhetoric, visuals, colours, ways in which consumers were invited to imagine themselves, how the tests were delivered and what kinds of products were offered in association with the tests and how those products were described. Companies had slightly different strategies in this respect, pitching their products as lifestyle or less-serious medicine (see Table 2). Such stratified categories suggest possible shapes that the market between medicine and consumer culture may take. The nutrigenetic testing market also clearly borrowed ideas from and collaborated with related industry, such as the supplements industry. In addition, the practices created by the more medically oriented companies, such as offering phone consultations with physicians, were similar to those of companies marketing genetic tests for predisposition to disease, such as DNAdirect.

In this new area, many questions remain. For example, whilst nutrigenetic expands biomedical jurisdiction into eating, it also operates in reverse, rendering a rarefied medical technology—genetic testing—consumer culture. Whilst (bio)medicalisation has been extensively studied, consumerization, i.e., rendering a medical technology consumer culture has rarely been investigated. It raises the question of, for example, whether and how does the meaning of genetic testing change for the consumer when it is sold adjacent to garden furniture online. How people understand and use lifestyle technologies clearly begs further research.

Furthermore, in 2009 Sciona and its distributors ceased selling nutrigenetic tests. This could be due to the drying of investment funds amidst global economic downturn or competition from new companies, such as 23andMe and deCODEme, selling whole genome scans. It is unclear if nutrigenetic testing as a business concept will survive, even if commercial genetic testing seems here to stay. We have here argued that the emergent categories between medicine and consumer culture, so well illustrated by nutrigenetic tests, are a broad phenomena, not limited to genetic testing. How the lifestyle market develops and perhaps consolidates into some consistent regulatory and marketing frameworks also calls for more research.

## Figures and Tables

**Table 1:** The model of service delivery, genetic tests and associated treatments offered by the nine online nutrigenetic testing companies analysed in 2008.

Company	Delivery	Genetic tests	Treatments
Genelex^a^http://www.genelex.com	Direct online Pre-test consultation with a “DNA Testing Consultant”	Nutritional genetic panel (Paternity, Ancestry, Drug reaction, Predictive)	Dietary advice Vitamin supplements

Genova Diagnostics/Genovations http://www.genovadiagnostics.com	Ordered through a physician or nutrition counsellor	‘CardioGenomicPlus’ ‘OsteoGenomic’ ‘EstroGenomic’ ‘DetoxiGenomic’ ‘ImmunoGenomic’ ‘NeuroGenomic’	Follow-up tests Dietary and medication advice

Genetic Health http://www.genetic-health.co.uk	Ordered via a telephone consultation with counsellor or other company staff member Post-test consultation with a doctor by phone or face-to-face	‘Nutrition Gene’ ‘Obesity, diabetes and weight loss’ (Premium Male, Premium Female, PharmacoGene)	Dietary advice Follow-up tests

Interleukin Genetics/Quixtar http://www.ilgenetics.com	Direct online	‘Gensona General Nutrition Genetic Test’ ‘Gensona Heart Health Genetic Test’	Dietary advice Special Nutrilite Heart Health Dietary Supplement Developing pharmaceutical, and nutritional products

Market America^a^http://www.marketamerica.com	Direct online	Gene SNP DNA analysis	Gene SNP custom genetic nutritional formula

Nutrigen^a^http://www.1-888-nutrigen.com	Direct online? With a pre or post consultation with a “medical expert” With a consultation on “Carb cycling diet”	‘Comprehensive’ ‘Bone Health’ ‘Heart Health’ ‘Insulin Resistance’ ‘Inflammation’ ‘Antioxidant/Detoxification’	Dietary advice Supplement advice Carb Cycling Diet

Salugen http://www.salugen.com	Spas and wellness centers (phone number offered)	‘GenoTrim Test’ ‘SpaGen Test’	Special supplements GenoTrim weight management regimen SpaGen nutrition regimen

Sciona^a^http://www.sciona.com	Direct online Through special pharmacies and intermediaries	‘MyCellf (The Science of You)’ ‘MyCellf (DNA Fitness)’	Dietary advice Supplement advice Exercise advice

Suracell http://www.suracell.com	Direct online (with possible phone consultation) Medispas Physician's Offices Wellness Centers	‘Personal DNA Analysis’	Nutraceuticals: Bone and Joint Health Blood Sugar and Body Fat Control Heart and Vascular Health Detoxification and free radical control Optimal cell function

a Indicates the company was no longer offering nutrigenetic tests in 2009.

**Table 2:** Emerging frames or models for genetic testing.
